# The simplified Kirchhoff network model (SKNM): a cell-based reaction–diffusion model of excitable tissue

**DOI:** 10.1038/s41598-023-43444-9

**Published:** 2023-09-30

**Authors:** Karoline Horgmo Jæger, Aslak Tveito

**Affiliations:** Simula Research Laboratory, Oslo, Norway

**Keywords:** Computational biophysics, Computer modelling, Numerical simulations, Biophysics

## Abstract

Cell-based models of excitable tissues offer the advantage of cell-level precision, which cannot be achieved using traditional homogenized electrophysiological models. However, this enhanced accuracy comes at the cost of increased computational demands, necessitating the development of efficient cell-based models. The widely-accepted bidomain model serves as the standard in computational cardiac electrophysiology, and under certain anisotropy ratio conditions, it is well known that it can be reduced to the simpler monodomain model. Recently, the Kirchhoff Network Model (KNM) was developed as a cell-based counterpart to the bidomain model. In this paper, we aim to demonstrate that KNM can be simplified using the same steps employed to derive the monodomain model from the bidomain model. We present the cell-based Simplified Kirchhoff Network Model (SKNM), which produces results closely aligned with those of KNM while requiring significantly less computational resources.

## Introduction

The bidomain model (BD)^1^ has become a valuable tool for understanding cardiac electrophysiology. It has been used, for instance, to study the effects of electrical defibrillation^2–4^, to identify drug effects from microphysiological systems^5,6^, and to personalize the identification of targets for ablation of atrial fibrillation^7^. A recent, comprehensive review of mathematical models of whole-heart electrophysiology can be found in Sung et al.^8^. In many cases, BD can be replaced by the somewhat simpler monodomain model (MD) which often yields very accurate approximations of the solution of BD, see, e.g., Franzone et al.^1^, Potse et al.^9^, and Sundnes et al.^10^. However, both BD and MD rely on homogenizations, and in this process the individual myocytes are removed from the model. The homogenized models (BD and MD) are therefore useful at the tissue level (mm), but runs into difficulties at finer scales (e.g., $\mu$m). Specifically, the homogenized models cannot be used to study electrophysiology in the vicinity of individual myocytes. Also, it is worth noticing that the myocytes do not re-appear in the mathematical model by mesh refinements; after homogenization, the myocytes are no longer part of the models regardless of the mesh resolution.

Because of the obvious need to enable analysis close to the myocytes, cell-based models have recently been developed^11–16^, applied^17–20^, and analyzed^21–26^. These models are accurate at the level of micrometers at the cost of significant increase in computational efforts needed to solve the equations.

In the recent paper^27^ we presented the Kirchhoff Network Model (KNM) which aims at balancing the need for cell-level accuracy with computational efficiency. The model represents every individual cell and the associated extracellular space of the tissue under consideration, but does not allow for spatial variation of parameters along the individual cell membrane or inside the cell. In other words, KNM allows the properties of individual cells to vary and can thus obtain cell-level accuracy, but cannot obtain sub-cellular accuracy. It was demonstrated in this paper^27^ that KNM required computational efforts comparable to BD and much smaller than the cell-based Extracellular-Membrane-Intracellular (EMI) model. For one example the CPU efforts of KNM was about 0.01% of the CPU efforts needed to solve the EMI model. This indicates that KNM can both achieve cell-level accuracy and be used in simulations with a large number of myocytes.

It is well known that BD reduces to the simpler MD under the assumptions of equal anisotropy ratios, see, e.g., Franzone et al.^1^, Potse et al.^9^, and Sundnes et al.^10^.  Furthermore, it is well known that the solutions of MD generally approximate the solutions of BD very well even if the assumption of equal anisotropy ratios do not hold, see Potse et al.^9^. Therefore, MD is commonly used as a reasonable approximation of BD. The derivation of MD based on BD is straightforward, and in the present report we will show that the same steps can be used to derive a simplified version of KNM. The simplified KNM will be referred to as SKNM. We will show that SKNM is significantly faster than KNM and we will discuss how well the solutions of SKNM approximate the solutions of KNM. Furthermore, we will compare the computational efforts needed to solve BD, MD, KNM and SKNM, and conclude that, when cell level accuracy is need, SKNM is the fastest model.

## Methods

Below, we will show numerical solutions using the bidomain model (BD), monodomain model (MD), the Kirchhoff network model (KNM) and the simplified Kirchhoff network model (SKNM). For readability, we repeat the formulation of BD, MD and KNM, and then we present the derivation of SKNM.

### The bidomain model (BD)

Let $C_m$ be the specific membrane capacitance (in μF/cm$^2$), $\chi$ be the membrane area to volume ratio (in cm$^{-1}$), $M_i$ and $M_e$ be intracellular and extracellular BD conductivity tensors (in mS/cm), $I_{\textrm{ion}}$ be the current density at the cell membrane (in μA/cm$^2$) and *F* be a function governing the dynamics a number of state variables *s*, modeling the membrane dynamics. Then, the bidomain model (see, e.g., Franzone et al.^1^ and Sundnes et al.^10^) reads

1 $$\begin{aligned} C_m\frac{\partial v}{\partial t}&= \chi ^{-1}\left( \nabla \cdot \left( M_i\nabla v\right) + \nabla \cdot \left( M_i\nabla u_e\right) \right) - I_{\textrm{ion}}(s,v), \end{aligned}$$

2 $$\begin{aligned} 0&= \nabla \cdot \left( M_i\nabla v\right) + \nabla \cdot \left( (M_i + M_e)\nabla u_e\right) , \end{aligned}$$

3 $$\begin{aligned} \frac{ds}{dt}&= F(s,v), \end{aligned}$$

where *v* and $u_e$ are the unknown functions to be found, representing the membrane potential and extracellular potential, respectively (both in mV). Here, we consider the two dimensional (2D) version of BD with diagonal conductivity tensors,

4 $$\begin{aligned} M_i&= \begin{pmatrix}M_i^x &{} 0\\ 0 &{} M_i^y\end{pmatrix},&M_e&= \begin{pmatrix}M_e^x &{} 0 \\ 0 &{} M_e^y\end{pmatrix}, \end{aligned}$$

where $M_i^x$ and $M_e^x$ are the BD conductivities in the *x*-direction, and $M_i^y$ and $M_e^y$ are the BD conductivities in the *y*-direction.

### The Kirchhoff network model (KNM)

**Figure 1:**
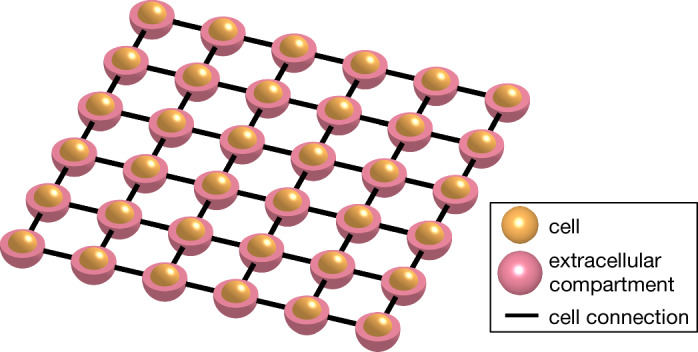
Illustration of the Kirchhoff network model (KNM). The model consists of a network of connected cells, each with a surrounding extracellular compartment. The model is derived by applying Kirchhoff’s current law for each cell and extracellular compartment.

KNM was introduced in Jæger et al.^27^ and consists of a collection of *N* cells with associated extracellular space, see Fig. 1. For cell number *k*, $u_i^k$ and $u_e^k$ are the potentials (in mV) of the intracellular and extracellular compartments, respectively. The associated membrane potential is given by $v_k=u_i^k-u_e^k$. We assume that cell number *k* is connected to a collection, $N_k$, of neighboring cells. The KNM then reads

5 $$\begin{aligned} C_m\frac{dv^k}{dt}&= \frac{1}{A_m^k}\sum _{j\in N_k}I_i^{j,k} -I_{\textrm{ion}}^k(v^k, s^k), \end{aligned}$$

6 $$\begin{aligned} 0&= \sum _{j\in N_k}I_i^{j,k} + \sum _{j\in N_k}I_e^{j,k}, \end{aligned}$$

7 $$\begin{aligned} \frac{ds^k}{dt}&= F_k(s^k,v^k). \end{aligned}$$

Here, $C_m$ is the specific membrane capacitance (in μF/cm$^2$), $A_m^k$ is the membrane area of cell *k* (in cm$^2$), $I_{\textrm{ion}}^k$ is the ionic current density through ion channels, pumps and exchangers on the membrane of cell *k* (in μA/cm$^2$) and $s^k$ is a set of additional state variables modeling the membrane dynamics of cell *k*.

#### Currents between neighboring compartments

If $j\in N_k$ (that is, if cell *j* is electrically coupled to cell *k*), the current between these cells is assumed to be given by

8 $$\begin{aligned} I_{i}^{j,k}&= G_i^{j,k}(u_i^{j}-u_i^{k}). \end{aligned}$$

Similarly, the current flow between neighboring extracellular compartments *j* and *k*, are given by

9 $$\begin{aligned} I_{e}^{j,k}&= G_e^{j,k}(u_e^{j}-u_e^{k}). \end{aligned}$$

Here, $G_i^{j,k}$ and $G_e^{j,k}$ are the total intracellular and extracellular conductances (in mS).

By inserting the currents (8) and (9) in the system (5)–(7), we can write the KNM system on the form

10 $$\begin{aligned} C_m\frac{dv^k}{dt}&= \frac{1}{A_m^k}\sum _{j\in N_k}G_i^{j,k}(u_i^{j}-u_i^{k}) -I_{\textrm{ion}}^k(v^k, s^k), \end{aligned}$$

11 $$\begin{aligned} 0&= \sum _{j\in N_k}G_i^{j,k}(u_i^{j}-u_i^{k}) + \sum _{j\in N_k}G_e^{j,k}(u_e^{j}-u_e^{k}) , \end{aligned}$$

12 $$\begin{aligned} \frac{ds^k}{dt}&= F_k(s^k,v^k). \end{aligned}$$

#### KNM equations mimicking the structure of the bidomain model

We we can now rewrite the KNM system to a form that is more similar to the BD equations above. In particular, (10) can be written on the form

$$\begin{aligned} C_m\frac{dv^k}{dt}&= \frac{1}{A_m^k}\sum _{j\in N_k}G_i^{j,k}(u_i^{j}-u_i^{k}) -I_{\textrm{ion}}^k(v^k, s^k), \\&=\frac{1}{A_m^k}\sum _{j\in N_k}G_i^{j,k}\left( (u_i^{j}-u_e^{j}) - (u_i^{k}-u_e^{k})+ (u_e^{j}-u_e^{k})\right) - I_{\textrm{ion}}^k(v^k, s^k), \\&=\frac{1}{A_m^k}\sum _{j\in N_k}G_i^{j,k}\left( (v^{j} - v^{k})+ (u_e^{j}-u_e^{k})\right) - I_{\textrm{ion}}^k(v^k, s^k). \end{aligned}$$

Similarly, the second equation (11) takes the form

$$\begin{aligned} 0&= \sum _{j\in N_k}G_i^{j,k}(u_i^{j}-u_i^{k}) + \sum _{j\in N_k}G_e^{j,k}(u_e^{j}-u_e^{k}) \\&= \sum _{j\in N_k}G_i^{j,k}\left( (u_i^{j}-u_e^{j}) -(u_i^{k}-u_e^{k}) +(u_e^{j}-u_e^{k}) \right) + \sum _{j\in N_k}G_e^{j,k}(u_e^{j}-u_e^{k}) \\&= \sum _{j\in N_k}G_i^{j,k}\left( (v^{j} -v^{k}) +(u_e^{j}-u_e^{k}) \right) + \sum _{j\in N_k}G_e^{j,k}(u_e^{j}-u_e^{k}) \\&= \sum _{j\in N_k}G_i^{j,k}\left( v^{j} -v^{k} \right) + \sum _{j\in N_k}(G_i^{j,k}+G_e^{j,k})(u_e^{j}-u_e^{k}). \end{aligned}$$

To summarize, the KNM system (5)–(7) can be written in the form

13 $$\begin{aligned} C_m\frac{dv^k}{dt}&= \frac{1}{A_m^k}\sum _{j\in N_k}\{G_i^{j,k} (v^{j} - v^{k})+ G_i^{j,k} (u_e^{j}-u_e^{k})\} - I_{\textrm{ion}}^k(v^k, s^k), \end{aligned}$$

14 $$\begin{aligned} 0&= \sum _{j\in N_k}G_i^{j,k}\left( v^{j} -v^{k} \right) + \sum _{j\in N_k}(G_i^{j,k}+G_e^{j,k})(u_e^{j}-u_e^{k}), \end{aligned}$$

15 $$\begin{aligned} \frac{ds^k}{dt}&= F_k(s^k,v^k). \end{aligned}$$

This form of the KNM system closely resembles the form (1)–(3) of the BD system. We will use this similarity to show that a simplified version of KNM can be derived in the same way as MD is derived from BD.

### Deriving MD from BD and the simplified KNM (SKNM) from KNM

#### The monodomain model (MD)

By assuming that the intracellular and extracellular conductivity tensors are related by a constant, BD simplifies considerably, see, e.g., Franzone et al.^1^ and Sundnes et al.^10^. To demonstrate this, we assume that there is a constant $\lambda$ such that

16 $$\begin{aligned} M_e=\lambda M_i. \end{aligned}$$

By using this assumption, it follows that

17 $$\begin{aligned} (M_i+M_e)\nabla u_e = (1+\lambda )M_i\nabla u_e, \end{aligned}$$

and inserting this in (2), we get

18 $$\begin{aligned} \nabla \cdot \left( M_i\nabla u_e\right) =-\frac{1}{1+\lambda }\nabla \cdot \left( M_i\nabla v\right) . \end{aligned}$$

By inserting this into (1), we obtain the monodomain model,

19 $$\begin{aligned} C_m\frac{\partial v}{\partial t}&= \frac{\lambda }{\chi (1+\lambda )} \nabla \cdot \left( M_i\nabla v\right) - I_{\textrm{ion}}(s,v), \end{aligned}$$

20 $$\begin{aligned} \frac{ds}{dt}&= F(s,v). \end{aligned}$$

#### The simplified KNM (SKNM)

In order to derive a similarly simplified version of KNM, we follow the steps above and start by assuming that there exists a constant $\lambda$ such that

21 $$\begin{aligned} G_e^{j,k}=\lambda G_i^{j,k}. \end{aligned}$$

From this assumption it follows that

22 $$\begin{aligned} (G_i^{j,k}+G_e^{j,k})(u_e^j-u_e^k) = (1+\lambda )G_i^{j,k}(u_e^j-u_e^k) \end{aligned}$$

and inserting this in (14), we get

23 $$\begin{aligned} \sum _{j\in N_k}G_i^{j,k}(u_e^{j}-u_e^{k})=-\frac{1}{1+\lambda }\sum _{j\in N_k}G_i^{j,k}\left( v^{j} -v^{k} \right) . \end{aligned}$$

By inserting this into (13), we can formulate the SKNM as follows,

24 $$\begin{aligned} C_m\frac{dv^k}{dt}&= \frac{\lambda }{A^k_m(1+\lambda )}\sum _{j\in N_k}G_i^{j,k} (v^{j} - v^{k}) - I_{\textrm{ion}}^k(v^k, s^k), \end{aligned}$$

25 $$\begin{aligned} \frac{ds^k}{dt}&= F_k(s^k,v^k), \end{aligned}$$

which resembles the form of MD (19)-(20).

### Defining the KNM and SKNM parameters

The conductance parameters $G_i^{j,k}$ and $G_e^{j,k}$ in KNM are defined as in Jæger et al.^27^,

26 $$\begin{aligned} G_e^{j,k}&= \delta _e^{j,k}\frac{A_{j,k}\sigma _e}{l_{j,k}}, \end{aligned}$$

27 $$\begin{aligned} G_i^{j,k}&= \frac{1}{\frac{l_{j,k}}{\delta _i^{j,k}A_{j,k}\sigma _i} + \frac{1}{G_g^{j,k}}}, \end{aligned}$$

where $\delta _e^{j,k}$ (unitless) is the mean extracellular volume fraction of cell compartments *j* and *k*, and $\delta _i^{j,k}=1-\delta _e^{j,k}$ (unitless) is the associated intracellular volume fraction. Furthermore $A_{j,k}$ (in cm$^2$) is the mean cross sectional area between the centers of cell compartments *j* and *k*, $l_{j,k}$ (in cm) is the distance between the centers, $G_g^{j,k}$ (in mS) is the conductance through the gap junctions connecting cells *j* and *k*, and $\sigma _i$ and $\sigma _e$ (both in mS/cm) are the conductivities of the intracellular and extracellular spaces, respectively. The derivation of the definitions (26)–(27) is described in the Supplementary Information.

#### Defining $\lambda$ in SKNM based on KNM parameters

Considering (26) and (27), the assumption

28 $$\begin{aligned} G_e^{j,k}=\lambda G_i^{j,k} \end{aligned}$$

for a constant value of $\lambda$ clearly does not hold in general. Below, we will consider cases where the gap junction resistances ($G_g^{j,k}$) vary randomly while keeping the extracellular conductance constant, and in such cases, the assumption (28) is clearly violated. In addition, we will consider cases of anisotropic cell geometry, also violating (28). However, as shown in Potse et al.^9^, MD often serves as a very good approximation of BD even if the MD assumption does not hold.

In order to define $\lambda$ in cases where (28) is violated, we seek a weighted least square approximation. To this end, we define the function

29 $$\begin{aligned} F(\lambda )= \sum _{j,k} \left( (G_e^{j,k}-\lambda G_i^{j,k} )\frac{l_{j,k}}{A_{j,k}} \right) ^2, \end{aligned}$$

whose minimum is given by

30 $$\begin{aligned} \lambda = \frac{ \sum _{j,k}G_e^{j,k}G_i^{j,k} \frac{l^2_{j,k}}{A^2_{j,k}} }{ \sum _{j,k} (G_i^{j,k})^2 \frac{l^2_{j,k}}{A^2_{j,k}} }. \end{aligned}$$

#### Defining $\lambda$ in MD based on BD parameters

In order to define the MD parameter $\lambda$ from the BD parameters, we similarly seek a least squares approximation, and define

31 $$\begin{aligned} F(\lambda ) = \frac{1}{2}\int _{\Omega }\left( (M_e^x-\lambda M_i^x)^2 + (M_e^y-\lambda M_e^y)^2\right) \,dS, \end{aligned}$$

where $\Omega$ is the computational domain and $M_i^x$, $M_i^y$, $M_e^x$ and $M_e^y$ are components of the BD conductivity tensors as defined in (4). In our computations, we use the minimum of (31),

32 $$\begin{aligned} \lambda&= \frac{\int _{\Omega }M_e^xM_i^x\,dS + \int _{\Omega }M_e^yM_i^y\,dS}{\int _{\Omega }(M_i^x)^2\,dS + \int _{\Omega }(M_i^y)^2\,dS}, \end{aligned}$$

to define $\lambda$ for MD.

### Numerical methods and software

All simulations reported in this paper are performed in C++ using the MFEM library for finite element methods^28,29^. The system of equations for each model is solved using standard operator splitting of the non-linear membrane system and the remaining linear system (see, e.g., Sundnes et al.^30^). The non-linear membrane equations are solved using the first-order Rush-Larsen method^31,32^ with code generated using the Gotran code generator^33^ and applying OpenMP parallelization^34^. The linear systems are solved using BiCGSTAB. For KNM and BD, we use a standard block Jacobi preconditioner^35^, whereas no preconditioner is used for SKNM and MD. We use meshes generated by Gmsh^36^ with a target mesh size of $\Delta x = 10\;$ μm and a time step of $\Delta t = 0.01$ ms for the BD and MD simulations. For KNM and SKNM, we use $\Delta t = 0.02$ ms. The choice of discretization parameters are based on convergence investigations reported in the Supplementary Information. All computations are run on a Dell Precision 3640 Tower with an Intel Core processor (i9-10900K, 3.7 GHz/5.4 GHz) with ten kernels with two threads each.

### Simulation set-up and parameter values

We consider two examples of potential areas of application for KNM and SKNM. The first example is a collection of human induced pluripotent stem cell-derived cardiomyocytes (hiPSC-CMs) and the second example is a collection of pancreatic $\beta$ cells. The model parameterizations for the two cases are described below.

#### Collection of hiPSC-CMs

In the simulations of hiPSC-CMs we consider a sheet of 40$\times$40 connected hiPSC-CMs, unless otherwise stated. The cells are assumed to have a surface area of $A_m=1.8\times 10^{-5}$ cm$^2$, and a volume of about 4 pL, based on measurements from Hwang et al.^37^. In the default case, we assume that each cell extends 16 μm in each spatial direction, and we assume that the default extracellular volume fraction is $\delta _e=0.2$. More specifically, we let $l_x=l_y=16\;$ μm and $l_z=(1+\delta _e)\cdot 16\;$ μm. The membrane dynamics (*F* and $I_{\textrm{ion}}$) are modeled using the wild-type hiPSC-CM model from Jæger et al.^38^. The default gap junction conductance, $G_g$, is set to $2\times 10^{-4}$ mS, in order for the conduction velocity to be about 4 cm/s, motivated by conduction velocities observed in sheets of hiPSC-CMs in Kadota et al.^39^, Kawatou et al.^40^, and Shinnawi et al.^41^.

#### Collection of pancreatic $\beta$ cells

In the simulations of a collection of pancreatic $\beta$ cells, we consider a sheet of 15$\times$15 cells. The cells are assumed to be shaped as spheres with a diameter of $l_x=l_y=l_z=13\;$ μm, based on Félix-Martínez et al.^42^ and Camunas-Soler et al.^43^. The membrane surface area is $A_m = 5.3\times 10^{-6}$ cm$^2$, and the default extracellular volume fraction is $\delta _e=0.5$. The membrane dynamics (*F* and $I_{\textrm{ion}}$) are modeled using the phantom bursting $\beta$ cell model from Bertram and Sherman^44,45^. The default gap junction conductance, $G_g$, is set to $2\times 10^{-7}$ mS, based on Loppini et al.^46^.

#### Boundary conditions

In the BD and KNM simulations, we apply homogenous Neumann boundary conditions for $u_e$ on the entire boundary, except in the lower left corner of the domain, where we apply a homogenous Dirichlet boundary condition for $u_e$ to ensure a unique solution of the system. Furthermore, we apply homogenous Neumann boundary conditions for *v* in all simulations. Note that since the extracellular potential is eliminated from the system in MD and SKNM, we only need to specify boundary conditions for the membrane potential for these models.

### Parameter variations

In order to investigate how well SKNM approximates KNM in cases where the assumption (21) does not hold, we consider some selections of parameter variations described below.

#### Varying the geometrical anisotropy of hiPSC-CMs

One case in which the assumption (21) may not hold is when the cell geometry is anisotropic. The shape of hiPSC-CMs have been reported to vary greatly, ranging from circular to elongated to triangular^37^. In our default case, we consider cells with the same extension in all spatial directions. However, we will also consider some cases of more elongated cells. More specifically, we introduce the cell length to width ratio, $\alpha$, defined such that $l_x=\alpha l_y$ and increase $\alpha$ gradually from the default value of 1 to a value of 4 while maintaining an intracellular cell volume of approximately 4 pL. More specifically, we set $l_y \approx \root 3 \of {\frac{4000\;\mu \textrm{m}^3}{\alpha }}$, $l_x = \alpha l_y$, and $l_z=(1+\delta _e)\cdot l_y$, where $\delta _e$ is the extracellular volume fraction. Note that the $\approx$ is used to indicate that the value of $l_y$ is rounded to the nearest 0.5 μm.

#### Inhomogeneous gap junction cell connections in KNM and SKNM

Another case in which the assumption (21) will not hold is if the gap junction conductance, $G_g^{j,k}$, varies randomly for each cell connection (*j*, *k*). In that case, $G_e^{j,k}$ is the same for all cell connections, whereas $G_i^{j,k}$ obtains different values for each connection (*j*, *k*). Therefore, no common $\lambda$ can be defined such that $G_e^{j,k}=\lambda G_i^{j,k}$.

We consider a gradual increase in the gap junction conductance variation by introducing the gap junction variation parameter, $\gamma$, that is allowed to vary from 0 to 1, where $\gamma =0$ represents no spatial variation and $\gamma =1$ represents the maximal degree of spatial gap junction variation. Furthermore, we draw a random number, $\theta _{j,k}$, between 0 and 1 for each cell connection and let

33 $$\begin{aligned} G_g^{j,k}&= \left( \theta _{j,k}(1-\gamma ) + (1-\theta _{j,k})(1+\gamma )\right) {\bar{G}}_g, \end{aligned}$$

where ${\bar{G}}_g$ is the default value for the gap junction conductance. To ease the comparison of the results, the same collection of random numbers, $\theta _{j,k}$, are used in all simulations and for both KNM and SKNM.

#### Inhomogeneous gap junction strength in BD and MD

 In BD and MD simulations, the individual cell connections are not as straightforwardly present in the model as in KNM and SKNM. However, in order to set up a similar test case for BD and MD, we let the value of $G_g$ vary in the same manner as for KNM and SKNM (following (33)) in different areas of the domain. More specifically, the domain is separated into areas of the size corresponding to one cell, and the value of $G_g$ is set up to vary in each such area. The bidomain conductivity tensors were set up as described in Jæger and Tveito^25^, i.e., by

34 $$\begin{aligned} M_i^x&= \frac{\delta _i \sigma _i}{1+\delta _i\sigma _i\frac{l_yl_z}{l_xG_g}}, \end{aligned}$$

35 $$\begin{aligned} M_i^y&= \frac{\delta _i \sigma _i}{1+\delta _i\sigma _i\frac{l_xl_z}{l_yG_g}}, \end{aligned}$$

36 $$\begin{aligned} M_e^x&= M_e^y = \delta _e\sigma _e, \end{aligned}$$

and we observe that when $G_g$ varies in space so does $M_i^x$ and $M_i^y$, whereas $M_e^x$ and $M_e^y$ remains constant.

#### Varying the extracellular volume fraction

In order to investigate how the differences and similarities between KNM and SKNM depend on the amount of extracellular space in the tissue, we consider four different values of the extracellular volume fraction, $\delta _e=0.5$, $\delta _e=0.2$, $\delta _e=0.1$, and $\delta _e=0.02$, corresponding to 50%, 20%, 10% and 2% extracellular space. The intracellular volume fraction is correspondingly updated so that $\delta _i+\delta _e=1$. It could be noted here that if the cells are shaped as spheres with radius *r* and organized in a structured manner, the intracellular volume is $\frac{4\pi }{3}r^3$, whereas the volume containing both a cell and an associated part of the extracellular domain could be $(2r)^3$. In that case, the intracellular volume fraction is $\delta _i\approx 0.5$, which also gives $\delta _e\approx 0.5$. Furthermore, in rat, rabbit and dog cardiac tissue, the extracellular volume has been estimated to be about 10–25% of the tissue volume^47–49^. The case of 2% extracellular space could therefore be regarded as a relatively extreme case, included mostly to illuminate the potential for model differences between KNM and SKNM.

### Definition of conduction velocity (CV)

#### hiPSC-CMs

In order to compute the conduction velocity (CV) for the collection of hiPSC-CMs we stimulate an area corresponding to the center 10 cells on the left boundary of the domain by a stimulus current of strength $20\;$ μA/cm$^2$. Then, the CV is computed by recording the two points in time, $t_a$ and $t_b$, when the membrane potential crosses a threshold value of $-20$ mV in the two cells *a* and *b* defined as cells number 10 and 35 in the *x*-direction and in the center of the domain in the *y*-direction. The CV is defined as

37 $$\begin{aligned} \textrm{CV}&= \frac{x_b-x_a}{t_b-t_a}, \end{aligned}$$

where $x_a$ and $x_b$ are the spatial *x* coordinates of the center of cells *a* and *b*, respectively.

#### $\beta$ cells

For the collection of pancreatic $\beta$ cells, we stimulate the center 5 cells on the left boundary of the domain, by setting the value of $g_{\textrm{KATP}}$ in the membrane model to half of its default value. This immediately starts rapid depolarization of the cells with a lower value of $g_{\textrm{KATP}}$ and initiates an excitation wave originating from these cells. Without this stimulation, rapid deploarization would not occur before about 100 seconds after the simulation started for the applied initial conditions and the default value of $g_{\textrm{KATP}}$. The CV is computed using (37), where cells *a* and *b* are cells number 5 and 13 in the *x*-direction and in the center of the domain in the *y*-direction, and $t_a$ and $t_b$ are the points in time when the membrane potential in these points crosses the threshold value of $-\,50$ mV.

## Results

In this section, we compare the results of SKNM to those obtained from KNM, first for a collection of hiPSC-CMs, and then for a collection of pancreatic $\beta$ cells. We also compare the CPU efforts required to run simulations of the two models. In addition, we perform BD and MD simulations to observe how the differences and similarities between SKNM and KNM compares to the differences and similarities between MD and BD.

### KNM and SKNM for a collection of hiPSC-CMs

**Figure 2:**
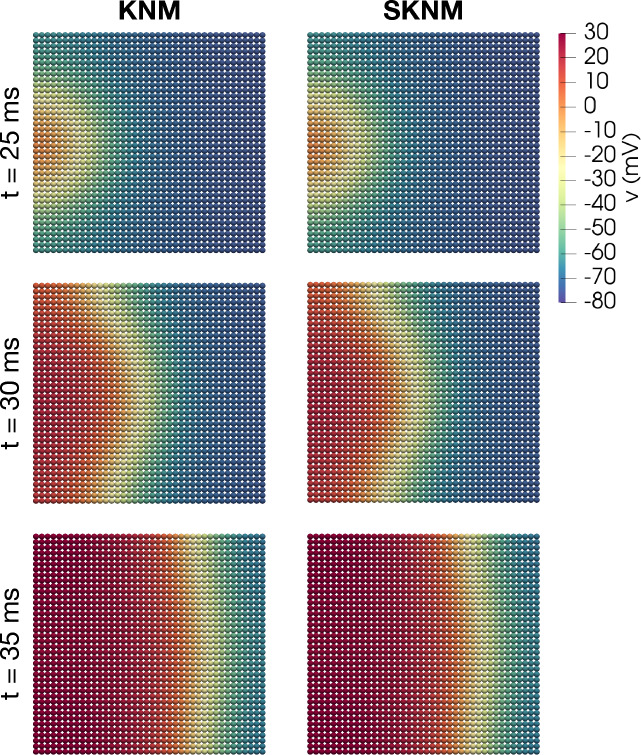
Traveling wave solutions of KNM and SKNM for the default model set-up for a collection of $40\times 40$ hiPSC-CMs. The figure displays snapshots of the membrane potential solution at three points in time during the simulation. In this case, the SKNM assumption (21) holds, and the solutions of the two models appears to be identical.

We first run KNM and SKNM simulations of a sheet of hiPSC-CMs. Figure 2 shows snapshots of the KNM and SKNM membrane potential solutions for this cell collection at three points in time. We observe that a traveling wave solution is generated moving from the left to the right side of the domain. Furthermore, we observe that the KNM and SKNM solutions appear to be identical. This is as expected because for this default case, the SKNM assumption (21) holds, and SKNM should be equivalent to KNM and provide the same solutions. However, the SKNM assumption (21) is not expected to hold in all cases, and we will now compare the KNM and SKNM solutions in some cases when the assumption is violated.

#### Comparison of KNM and SKNM solutions for hiPSC-CMs with anisotropic cell geometry

**Figure 3:**
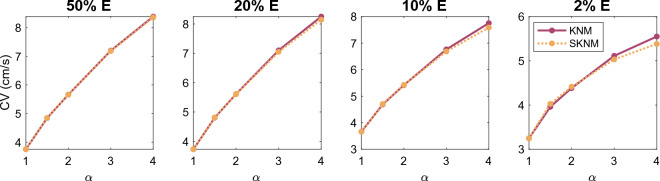
Comparison of the conduction velocity (CV) computed using KNM and SKNM for a collection of hiPSC-CMs with an increasing cell length to width ratio, $\alpha$ (see the “Parameter variations” section). We consider the cases of 50%, 20%, 10%, and 2% extracellular space as indicated in the panel titles.

We consider the case of an anisotropic cell geometry as explained in the  “Parameter variations” section. Figure 3 reports the CV computed for SKNM and KNM as the cell length to width ratio, $\alpha$, is increased from the default value of 1 (corresponding to equal cell length and width) to the value of 4 (corresponding to a cell length that is 4 times longer than the cell width). Furthermore, we consider the cases of 50%, 20%, 10% or 2% extracellular volume to investigate how the similarities or differences between SKNM and KNM depends on the extracellular volume fraction. In Fig. 3, we observe that even though the SKNM assumption (21) only holds for $\alpha = 1$, the CVs computed using SKNM seem to provide very good approximations for the CVs computed using KNM.

#### Comparison of KNM and SKNM solutions for hiPSC-CMs with inhomogeneous gap junction coupling

Next, we consider the case of an inhomogeneous gap junction coupling between cells. In this case, $G_e^{j,k}$ is the same for all cell connections, but $G_i^{j,k}$ given by (27) will vary for each cell connection. Since $G_e^{j,k}$ is constant and $G_i^{j,k}$ varies, the assumption (21) cannot hold. The degree of variability is represented by the gap junction variation parameter, $\gamma$, as explained in the “Parameter variations” section. In short, $\gamma =0$ represents no spatial variation of the gap junction conductance and $\gamma =1$ represents a randomly varying gap junction conductance ranging from 0 to $2{\bar{G}}_g$, where ${\bar{G}}_g$ is the default gap junction conductance value.

**Figure 4:**
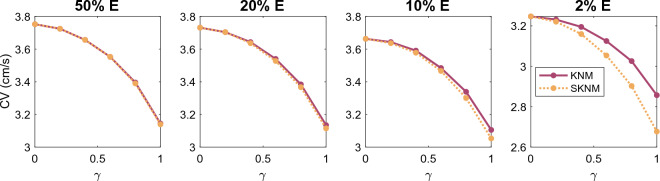
Comparison of the conduction velocity (CV) computed using KNM and SKNM for a collection of hiPSC-CMs with an increasing gap junction conductance variation, $\gamma$ (see the “Parameter variations” section). We consider the cases of 50%, 20%, 10%, and 2% extracellular space as indicated in the panel titles.

In Figure 4, we compare the CV computed using the KNM and SKNM solutions as the gap junction conductance variation $\gamma$ is increased for the four different values of the extracellular volume percentage. We observe that the KNM and SKNM solutions appear to be very similar in most cases. The exception is when the extracellular volume is very small. In that case, there is a considerable difference between the CVs of the two models and the difference increases as the gap junction variation, $\gamma$, is increased.

#### Sources of difference between KNM and SKNM

In Fig. 4, we observed that the difference between KNM and SKNM increased as the gap junction conductance variation, $\gamma$, was increased and as the size of the extracellular space, $\delta _e$, was decreased. In order to examine the potential sources of these effects, we recall the SKNM assumption, (21)

38 $$\begin{aligned} {{G_e^{j,k} = \lambda G_i^{j,k},}} \end{aligned}$$

which was used to get (22)

39 $$\begin{aligned} {{(G_i^{j,k}+G_e^{j,k})(u_e^j-u_e^k) = (1+\lambda )G_i^{j,k}(u_e^j-u_e^k),}} \end{aligned}$$

which was inserted into the KNM equations to derive SKNM. In the case when (38) is not fulfilled, neither is (39), and introducing (39) into KNM represents an approximation. The error of this approximation for each cell connection *j*, *k* is given by

40 $$\begin{aligned} {{E_{j,k}}}&{{= |(G_e^{j,k}-\lambda G_i^{j,k})(u_e^j-u_e^k)|.}} \end{aligned}$$

**Figure 5:**
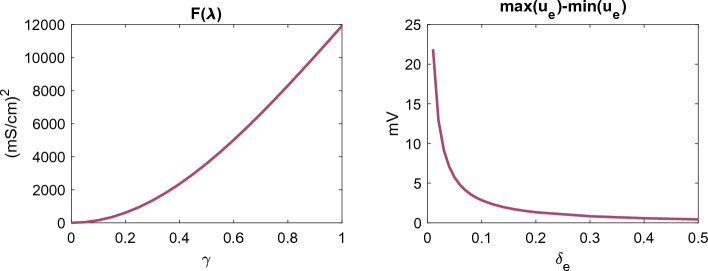
Sources of difference between KNM and SKNM. The left panel illustrates that the first factor of the SKNM approximation (40) increases as the gap junction conductance variation, $\gamma$, increases, while the right panel illustrates that the second factor of (40) decreases as the extracellular volume fraction, $\delta _e$ increases, explaining the SKNM and KNM differences observed in Fig. 4. In the left panel, the extracellular space is 20% ($\delta _e=0.2$) and $F(\lambda )$ and $\lambda$ are defined as in (29) and (30), respectively. In the right panel, $\gamma =0.5$.

As explained above, when gap junction conductance variation, $\gamma > 0$, is introduced, the assumption (39) cannot hold for all *j*, *k* since $G_e^{j,k}$ is constant in space while $G_i^{j,k}$ varies. This makes the first factor in (40) non-zero. Furthermore, in Fig. 5, we illustrate that the size of this factor increases as the value of $\gamma$ increases, illustrated by showing that the minimum value of $F(\lambda )$ defined in (29) to measure the difference between $G_e^{j,k}$ and $\lambda G_i^{j,k}$ increases for an increased value of $\gamma$. This indicates that the first term in (40) and thus the difference between SKNM and KNM should increase when $\gamma$ is increased, as observed in Fig. 4.

In addition, Fig. 5 shows the difference between the maximum and minimum values of the extracellular potential, $u_e$, during the simulation for different values of the extracellular volume fraction. We observe that this difference decreases as the size of the extracellular space increases, indicating that the second factor in (40) decreases. This indicates that the difference between KNM and SKNM might decrease for an increased size of the extracellular space, consistent with what is observed in Fig. 4.

#### Comparison of CPU efforts for KNM and SKNM for hiPSC-CMs

In Table 1, we report the CPU time required for a 10 ms long simulation of hiPSC-CMs using KNM and SKNM. The table reports the CPU time as the number of cells, *N*, included in the simulation increases, as well as the required CPU time per cell. We observe that the CPU time per cell appears to be close to constant for SKNM. For KNM, the CPU time per cell increases as the number of cells increases, and the CPU time appears to be approximately proportional to $N^{1.5}$ (see the fourth column of Table 1). Furthermore, we observe that the CPU time required for SKNM is significantly shorter than the time required for KNM. For instance, for 50,625 cells, the required CPU time is about 90 times longer for KNM than for SKNM.

**Table 1:** CPU time for 10 ms simulation of KNM and SKNM for a collection of *N* hiPSC-CMs.

*N*	$T_{\textrm{KNM}}$ (sec)	$T_{\textrm{KNM}}/N$ (ms)	$T_{\textrm{KNM}}/N^{1.5}$ (ms)	$T_{\textrm{SKNM}}$ (sec)	$T_{\textrm{SKNM}}/N$ (ms)
400	0.4	0.9	0.04	0.04	0.11
1600	1.9	1.2	0.03	0.15	0.09
6400	33.0	5.2	0.06	0.88	0.14
25,600	231.7	9.1	0.06	3.43	0.13
50,625	600.5	11.9	0.05	6.68	0.13

### BD and MD for a collection of hiPSC-CMs

As SKNM was derived from KNM in the same manner as MD can be derived from BD, it is interesting to observe whether the differences and similarities in model solutions (Figs. 3 and 4) and CPU requirements (Table 1) between SKNM and KNM are similar to those between BD and MD. In this subsection we therefore perform BD and MD simulations similar to those performed for KNM and SKNM in the previous subsection.

**Figure 6:**
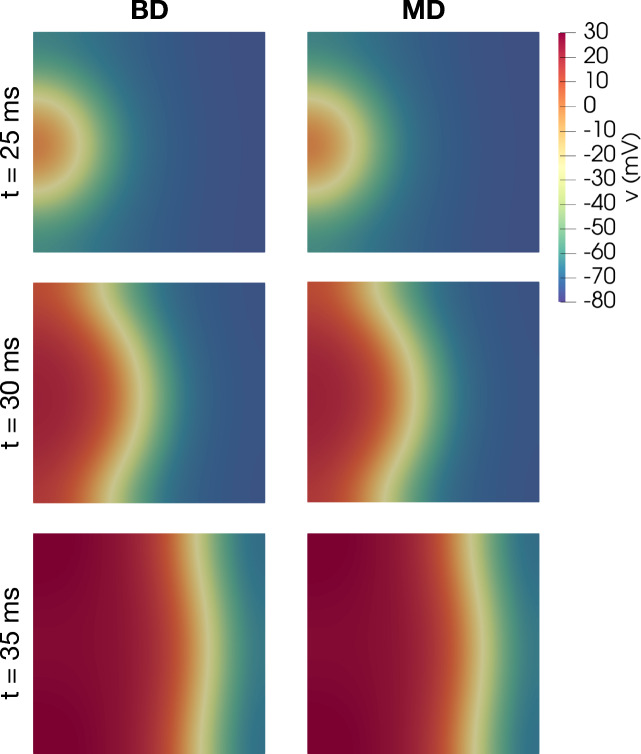
Traveling wave solutions of BD and MD for the default model set-up for a collection of hiPSC-CMs. The figure displays snapshots of the membrane potential solution at three points in time during the simulation. In this case, the MD assumption (21) holds, and the solutions of the two models appears to be identical.

First, in Fig. 6, we show snapshots of the BD and MD solutions for the default model set-up for a collection of hiPSC-CMs, similar to what was shown for KNM and SKNM in Fig. 2. As expected, the MD and BD solutions appear to be identical in this case when the MD assumption (16) holds.

#### Comparison of BD and MD solutions for hiPSC-CMs

**Figure 7:**
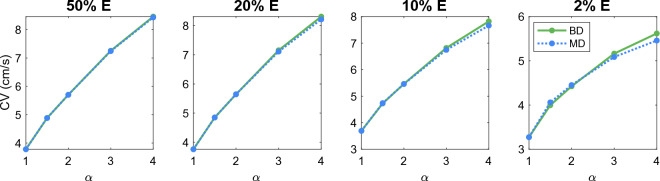
Comparison of the conduction velocity (CV) computed using BD and MD for a collection of hiPSC-CMs with an increasing cell length to width ratio, $\alpha$ (see the “Parameter variations” section). We consider the cases of 50%, 20%, 10%, and 2% extracellular space as indicated in the panel titles.

**Figure 8:**
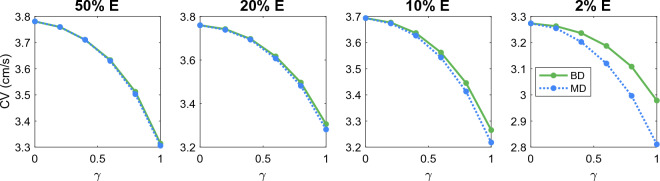
Comparison of the conduction velocity (CV) computed using BD and MD for a collection of hiPSC-CMs with an increasing gap junction conductance variation, $\gamma$ (see the “Parameter variations” section). We consider the cases of 50%, 20%, 10%, and 2% extracellular space as indicated in the panel titles.

In Figs. 3 and 4, we observed that the CV computed using SKNM seemed to approximate that computed using KNM very well in most cases, but for a very small extracellular volume fraction and a large degree of spatial variation in the gap junction coupling, some significant differences were observed. In Figs. 7 and 8, we report similar comparisons for the bidomain model (BD) and the monodomain model (MD). Comparing Figs. 3 and 7 and comparing Figs. 4 and 8 we observe that the differences and similarities between SKNM and KNM resemble those between MD and BD. In particular, MD seems to approximate BD very well for an anisotropic cell geometry, but a considerable difference between the two models is observed for a large degree of spatial variation in the gap junction coupling and a very small extracellular volume fraction.

#### Sources of difference between BD and MD

Like for KNM and SKNM, the difference between the BD and MD solutions appears to increase for an increased gap junction conductance variation, $\gamma$, and a decreased extracellular volume fraction, $\delta _e$. In order to examine these effects, we use the same approach as in the SKNM case and recall the monodomain assumption (16),

41 $$\begin{aligned} {{M_e=\lambda M_i,}} \end{aligned}$$

which was used to get (17)

42 $$\begin{aligned} {{(M_i+M_e)\nabla u_e = (1+\lambda )M_i\nabla u_e,}} \end{aligned}$$

which was inserted into BD to derive MD. In the case when (41) is not fulfilled, neither is (42), and introducing (42) into BD represents an approximation. The size of the error of this approximation is given by

43 $$\begin{aligned} {{E = \Vert (M_e-\lambda M_i)\nabla u_e\Vert .}} \end{aligned}$$

**Figure 9:**
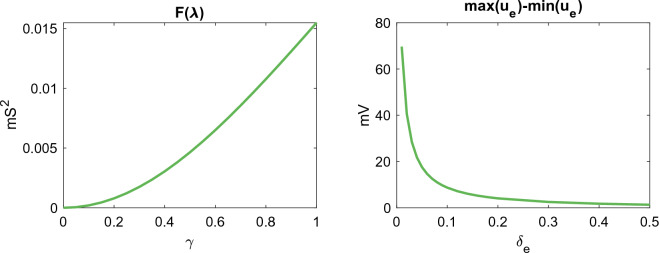
Sources of difference between BD and MD. The left panel illustrates that the first factor of the MD approximation (43) increases as the gap junction conductance variation, $\gamma$, increases, while the right panel illustrates that the second factor of (43) decreases as the extracellular volume fraction, $\delta _e$ increases, explaining the MD and BD differences observed in Fig. 8. In the left panel, the extracellular space is 20% ($\delta _e=0.2$) and $F(\lambda )$ and $\lambda$ are defined as in (31) and (32), respectively. In the right panel, $\gamma =0.5$.

Like for SKNM, when gap junction variation is introduced, the MD assumption (41) cannot hold everywhere because $M_e$ is constant in space while $M_i$ varies in space. This would make the first factor in (43) non-zero. Furthermore, Fig. 9 shows that the minimum value of $F(\lambda )$ increases as $\gamma$ is increased, indicating that the first factor in (43) and thus the difference between MD and BD should increase when $\gamma$ is increased, as observed in Fig. 8.

In addition, Fig. 9 shows the difference between the maximum and minimum values of the extracellular potential, $u_e$, during the simulation for different values of the extracellular volume fraction. We observe that this difference decreases as the size of the extracellular space increases, indicating that the second factor in (43) decreases. This indicates that the difference between MD and BD might decrease for a increased size of the extracellular space, consistent with what is observed in Fig. 8.

#### Comparison of CPU efforts for BD and MD for hiPSC-CMs

Table 2 shows the CPU times for a 10 ms simulation using BD and MD as the number of cells, *N*, included in the simulation increases. The increasing cell number is incorporated by adjusting the size of the spatial domain so that there is room for the considered number of cells. Like for KNM and SKNM, we observe that the CPU time for MD increases approximately linearly with the number of cells, whereas the time to solve BD increases as the number of cells increases by a rate of approximately $N^{1.5}$. Furthermore, the CPU time is considerably smaller for MD than for BD. For instance, for 50,625 cells, the CPU time is about 60 times longer for BD than for MD.

**Table 2:** CPU time for 10 ms simulation of BD and MD for a collection of *N* hiPSC-CMs.

*N*	$T_{\textrm{BD}}$ (sec)	$T_{\textrm{BD}}/N$ (ms)	$T_{\textrm{BD}}/N^{1.5}$ (ms)	$T_{\textrm{MD}}$ (sec)	$T_{\textrm{MD}}/N$ (ms)
400	18.6	46.5	2.3	2.5	6.3
1600	133.4	83.4	2.1	10.5	6.5
6400	874.9	136.7	1.7	35.1	5.5
25,600	7484.5	292.4	1.8	140.0	5.5
50,625	18552.2	366.5	1.6	297.4	5.9

### KNM and SKNM for a collection of pancreatic $\beta$ cells

In addition to the collection of hiPSC-CMs considered so far, we will also apply KNM and SKNM for a second example application; a collection of pancreatic $\beta$ cells.

#### Comparison of KNM and SKNM solutions for $\beta$ cells with inhomogeneous gap junction coupling

**Figure 10:**
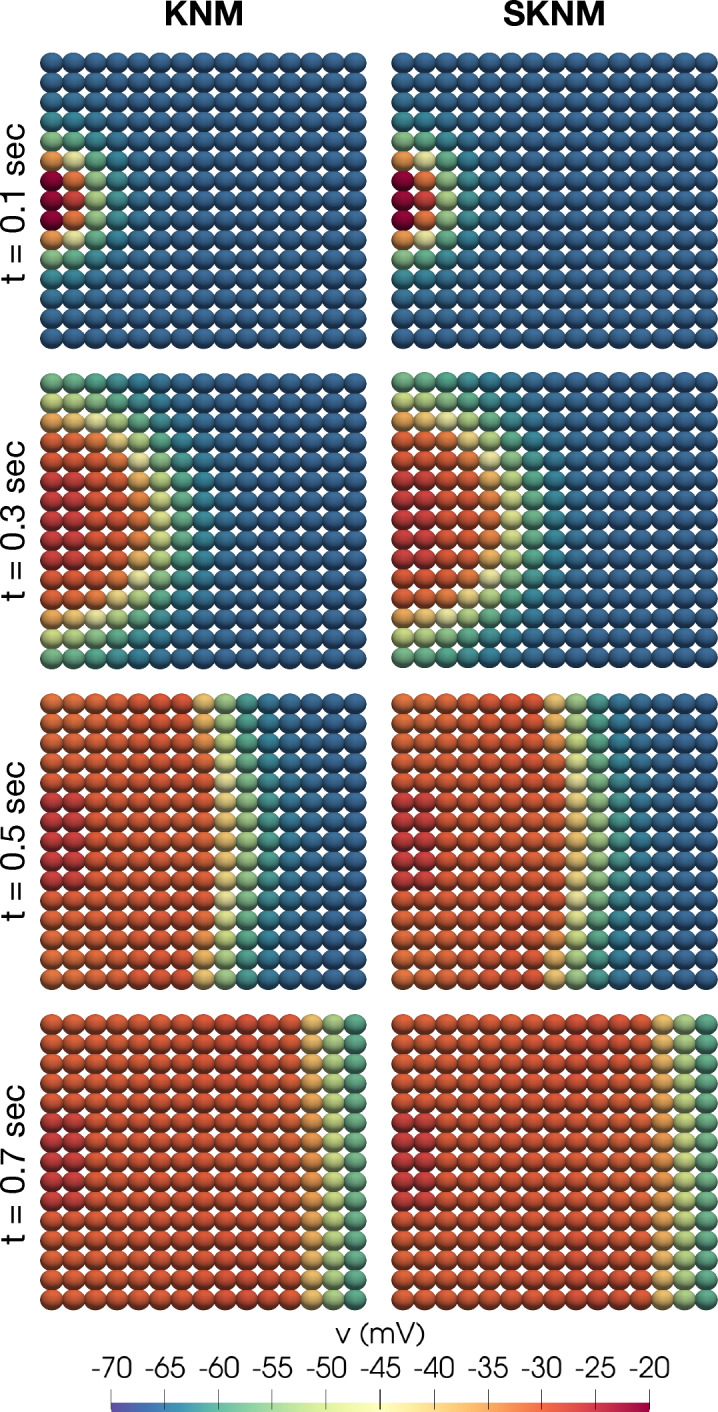
Snapshots of the membrane potential, *v*, computed using KNM and SKNM for the default pancreatic $\beta$ cell collection set-up.

**Figure 11:**
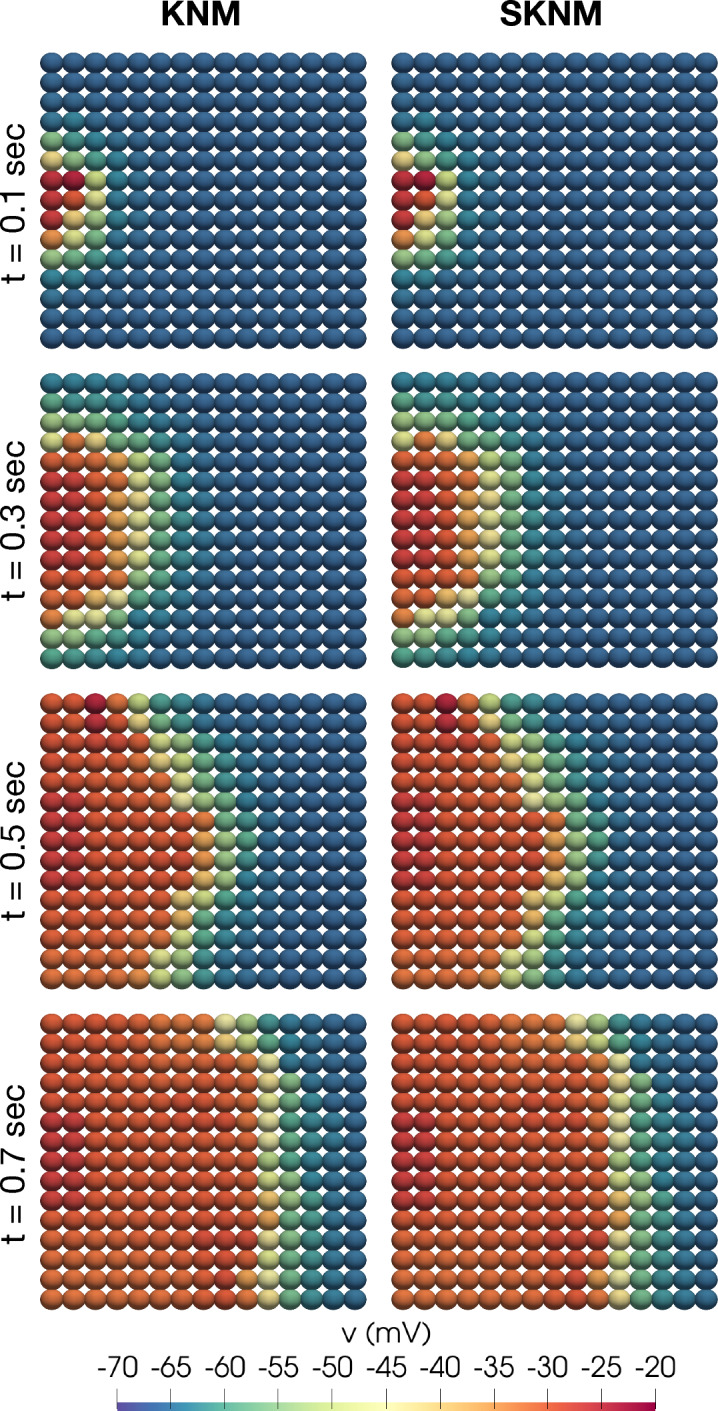
Snapshots of the membrane potential, *v*, computed using KNM and SKNM for a collection of $\beta$ cells with a variable gap junction coupling, $\gamma =1$ (see the “Parameter variations” section), and 2% extracellular volume.

In Fig. 10, we show snapshot solutions of an excitation wave traveling through a collection of $\beta$ cells, computed using KNM and SKNM. In this case, all cell connections are the same and the SKNM assumption (21) holds. Therefore, we expect the KNM and SKNM solutions to be the same, and this is confirmed in the figure. In Fig. 11 we consider a similar example where the KNM assumption do not hold because $G_e^{j,k}$ is constant, but $G_i^{j,k}$ varies for each cell connection as described in the “Parameter variations” section. In addition, we have set the extracellular volume fraction to 2%, since this appeared to give the largest discrepancy between SKNM and KNM in Fig. 4. Nevertheless, the KNM and SKNM solutions appear to be very similar for this example as well.

**Figure 12:**
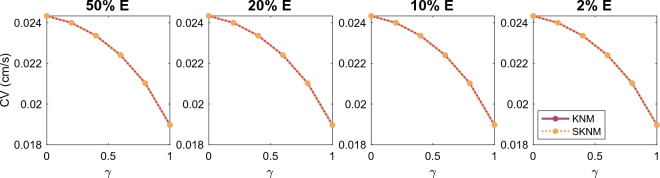
Comparison of the conduction velocity (CV) computed using KNM and SKNM for a collection of pancreatic $\beta$ cells with an increasing gap junction conductance variation, $\gamma$ (see the “Parameter variations” section). We consider the cases of 50%, 20%, 10%, and 2% extracellular space as indicated in the panel titles.

In Fig. 12, we have computed the conduction velocity for several choices of the gap junction variation, $\gamma$ (see the “Parameter variations” section), and the extracellular volume fraction like we did for hiPSC-CMs in Fig. 4. We observe that for the collection of $\beta$ cells, KNM and SKNM provide identical solutions in terms of conduction velocity. In fact, for the $\beta$ cell examples considered in this section, it turns out that a version of the SKNM based on a negligible extracellular potential also provides virtually identical solutions to KNM (see the Supplementary Information).

## Discussion

Our aim is to derive a fast and accurate model for small collections of excitable cells. Homogenized models like BD and MD are often considered to be reasonable in terms of computational demands, but lack representation of the very building block of the excitable tissue: the cell. On the other hand, the recently developed Extracellular-Membrane-Intracellular (EMI) model allows detailed representation of every cell at the expense of a very high computational cost. In the recent paper^27^, an alternative cell-based model was developed based on Kirchhoff’s current law. The model was referred to as the Kirchhoff Network Model (KNM) and allows representation of every cell, but not spatial representation of sub-cellular properties. Here, we have presented a simplification of KNM referred to as SKNM. The simplification follows the steps used to derive MD based on BD.

### Simulating microphysiological systems (MPS)

Traditionally, cardiomyocyte analysis has been conducted using animal cells. However, this approach to understanding human cardiomyocytes presents significant challenges due to the intrinsic differences between human and animal cells. The advent of human induced pluripotent stem cells (hiPSCs), created by reprogramming somatic cells, such as skin cells^50^, opens a substantial advancement in biological research^51^. These hiPSCs can be differentiated into cardiomyocytes (hiPSC-CMs), which can be examined within microphysiological systems, often referred to as organs on a chips^52,53^. Specifically, these systems hold considerable potential for detecting undesirable side effects of emerging drug compounds^54–56^.

To use measurements from an MPS for identifying potential side effects of a drug, it is essential to establish a precise mathematical model capturing the dynamics of hiPSC-CMs within the MPS. Several mathematical models have been developed to describe the action potential of hiPSC-CMs, see, e.g., Kernik et al.^57^, Paci et al.^58^, and Jæger et al.^58,59^ Additionally, the issue of modeling hiPSC-CMs as interconnected tissue has been addressed using the BD framework^6,60,61^. Within an MPS, the quantity of cardiomyocytes is typically relatively modest, ranging from thousands to millions^52,55,62^. The task of simulating these cells aligns well with the capabilities of KNM and SKNM. These models provide the flexibility of representing individual cells and are especially well-suited given the relatively low cell count when compared to whole animal or human hearts.

### The inverse problem of multi-well facilities

 Multi-well experimental facilities have recently been utilized to facilitate simultaneous measurements of hiPSC-CMs exposed to varying drugs at different dosages. In these facilities, each well houses several thousand cells. Rapid simulation of such cell collections is critical, as the simulation software will be employed to solve an inverse problem: given the measurements of the membrane potential and the cytosolic calcium concentration, deduce the properties of the ion channels^6,59,63^. Specifically, if a drug is introduced and it perturbs the membrane potential and the cytosolic calcium concentration, the inverse problem involves tracing back the source of these changes to the drug’s influence on the ion channels. This inverse problem is commonly resolved by minimizing a cost function iteratively, see, e.g., Jæger et al.^38^. Therefore, the simulation tool must be used repeatedly, making computational efficiency paramount.

### Computing efforts

Tables 1 and 2 present the CPU efforts required for KNM/SKNM and BD/MD, respectively. Analyzing this data, we estimate that the computational time needed to simulate a complete action potential of approximately 500 ms for 50,625 cells is 8.3 h for KNM, 5.6 minutes for SKNM, 10.7 days for BD, and 4.1 h for MD.

Clearly, if the simulator is employed iteratively for minimizing a cost function – a process that could entail numerous iteration – the computational efforts for all models except for SKNM would be prohibitively large. Consequently, SKNM emerges as the most promising alternative for such a task.

It should be noted that the CPU efforts depend on the computational set-up in terms of discretization parameters and solution methods. Here, we have used a much finer mesh than is common for BD and MD. Our discretization parameters are based on convergence analysis reported in the Supplementary Information. To reach convergence in the sense that the error is smaller than 2%, we need to have spatial resolution of about $\Delta x = 10\;$ μm. This is a much finer mesh than is often used in computational BD/MD analysis of large tissues. A mesh with $\Delta x = 0.25\;$mm is applied by many authors^64–67^. With such a resolution, almost 1,000 adult cardiomyocytes can be placed within each computational block^13^ and this is clearly too coarse for simulation of a few thousand hiPSC-CMs.

It should also be noted that the computational costs of both SKNM and MD are proportional to the number of cells under consideration, *N*, which is optimal. For both BD and KNM we found that the computational cost increased in proportion to $N^{1.5}$ which is not optimal. Since optimal *O*(*N*) solvers have been developed for BD^68–70^, it is reasonable to assume that it is possible to similarly improve the computational cost of KNM. This will be of importance in the case of large values of *N*.

Finally, it is worth emphasizing that our primary aim has not been to provide an exhaustive analysis of CPU efforts for each model, but rather to report our observations. A comprehensive comparison would necessitate a detailed examination of all implementation aspects, which we consider beyond the scope of this work. Our focus has been on presenting a cell-based model that strikes a balance between accuracy and computational efficiency.

### Simulation of large tissues

It should be noted that the considerations presented here holds for small cell collections. For both KNM and SKNM, the individual cells represent the only discretization in space, and simulation of large tissues requires the use of a very large number of cells. Under such conditions, the traditional BD and MD models are faster as these models can use a much coarser mesh than is defined by the cell size. The application of KNM and the faster SKNM should be considered in cases where it is of importance to represent the cells individually. Furthermore, if spatial representation at the sub-cellular level is needed, the EMI model^11–13^ should be applied.

### Pancreatic $\beta$ cells

The problem of simulating a collection of hiPSC-CMs is, in mathematical terms, very similar to simulating a collection of pancreatic $\beta$ cells, and we have demonstrated that the KNM and SKNM modeling approaches are applicable for pancreatic $\beta$ cells, see Figs. 10, 11 and 12. Furthermore, we found that using a version of SKNM where the extracellular potential is assumed to be negligible, SKNM($u_e=0$), provided a very good approximation of the full KNM set-up (see the Supplementary Information). This version of SKNM has been applied in a number of computational studies of collections of pancreatic $\beta$ cells, e.g.^71–76^. In these studies, an additional modeling simplification is often included by setting $G_i^{j,k} = G_g^{j,k}$. Recalling the formula for $G_i^{j,k}$ given in (27),

44 $$\begin{aligned} G_i^{j,k}&= \frac{1}{\frac{l_{j,k}}{\delta _i^{j,k}A_{j,k}\sigma _i} + \frac{1}{G_g^{j,k}}}, \end{aligned}$$

we observe that this simplification amounts to ignoring the first of the two terms in the denominator of (44). For the default parameters used for $\beta$ cells in our study, this first term, $\frac{l_{j,k}}{\delta _i^{j,k}A_{j,k}\sigma _i}$, is about 10,000 times smaller than the second term, $\frac{1}{G_g^{j,k}}$, so this additional simplification appears to be warranted in the $\beta$ cell case. For hiPSC-CMs, on the other hand, the second term is only about 6.5 times larger than the first term, suggesting that both terms should be included in the denominator of (44) for hiPSC-CMs. Moreover, as observed in the Supplementary Information, the assumption of a negligible extracellular potential only appears to be applicable for hiPSC-CMs if the extracellular volume percentage is quite large (e.g., 50%).

### Can the solutions of BD, MD, KNM and SKNM all coincide?

Because of the similarity of BD and KNM and of MD and SKNM, it is tempting to ask whether there are examples where the algebraic equations defining all four models can coincide completely. We demonstrate that this is indeed the case for a special case presented in the Supplementary Information. Note however, that the BD and KNM are fundamentally different in the sense that the cell is removed for the BD formulation whereas it is the very building block of the KNM approach. The same holds for MD and SKNM. The BD and MD models always require the choice of a specific numerical mesh whereas the cells define the spatial resolution of the KNM and the SKNM. Therefore, these models coincide only for very specific choices of meshes and parameters.

## Conclusion

In this study, we present the Simplified Kirchhoff Network Model (SKNM), designed as a computationally efficient approximation to the Kirchhoff Network Model (KNM). The SKNM’s theoretical basis is similar to the manner in which the monodomain model (MD) is derived from the bidomain model (BD). Similar to the KNM, the SKNM is constructed to represent each individual cell within the tissue. This feature enables the model to account for variations in membrane properties for individual cells and in the electrical coupling between adjacent cells.

### Supplementary Information

Supplementary Information.

## Data Availability

The data and code generated in this study are publicly available at Zenodo: 10.5281/zenodo.8340201^77^.
