# Supplementary material for: The simplified Kirchhoff network model (SKNM): a cell-based reaction–diffusion model of excitable tissue
Source: Sci Rep. 2023 Sep 30;13:16434. doi: 10.1038/s41598-023-43444-9 (PMC10542379; doi:10.1038/s41598-023-43444-9)
Supplement: Supplementary file 1 — Supplementary Information. [file 41598_2023_43444_MOESM1_ESM.pdf]

Supplementary Information

Contents

S1 Derivation of the KNM parameters **2**
S1.1 The extracellular conductance, $G_e^{j,k}$ 2
S1.2 The conductance between cells, $G_i^{j,k}$ 2

S2 Convergence of solutions **4**
S2.1 Computation of the maximal upstroke velocity 4
S2.2 Convergence of KNM and SKNM solutions for hiPSC-CMs . . 4
S2.3 Convergence of BD and MD solutions for hiPSC-CMs 5
S2.4 Convergence of KNM and SKNM for pancreatic β cells 6

S3 SKNM based on negligible extracellular potential **7**
S3.1 SKNM($u_e = 0$) for a collection of β-cells 7
S3.2 SKNM($u_e = 0$) for a collection of hiPSC-CMs 8

S4 A special case where the discretized BD and MD are equal to KNM and SKNM **9**
S4.1 Modeling setup 9
S4.2 Finite difference approximation of BD equal to KNM 9
S4.2.1 Rewriting the discretization in terms of general neighbors 10
S4.2.2 Boundary conditions 12
S4.2.3 Finite difference approximation of MD equal to SKNM 13
S4.3 Equal algebraic equations for KNM, SKNM, BD and MD . . . 13

S1 Derivation of the KNM parameters

In this section, we give an explanation for the definition of the KNM conductance parameters, $G_e^{j,k}$ and $G_i^{j,k}$.

S1.1 The extracellular conductance, $G_e^{j,k}$

The parameter $G_e^{j,k}$ represents the conductance of the electrical currents between the centers of the extracellular compartments surrounding cells j and k. In general, the conductance through a medium of length l (in cm), with conductivity σ (in mS/cm) and with cross-sectional area A (in cm^2) is given by [1]

$$G = \frac{A\sigma}{l}. \tag{S1}$$

In the special case of the current between the centers of two extracellular compartments, j and k, the distance, l is given by $l_{j,k}$ and the conductivity, σ, is given by the extracellular conductivity, σ_e. Furthermore, the cross-sectional area, A, of the extracellular compartments can be approximated by $\delta_e^{j,k} A_{j,k}$, where $\delta_e^{j,k}$ is the mean extracellular volume fraction of cell compartments j and k, and $A_{j,k}$ is the mean total cross-sectional area between the centers of compartments j and k including both the intracellular and extracellular parts of the compartments. Summing up, this gives

$$G_e^{j,k} = \delta_e^{j,k} \frac{A_{j,k}\sigma_e}{l_{j,k}}. \tag{S2}$$

S1.2 The conductance between cells, $G_i^{j,k}$

In order to model the conductance for the currents between connected cells, it is convenient to first consider the reciprocal of the conductance, i.e., the resistance. We assume that this resistance is given as the sum of the purely intracellular resistance ($R_c^{j,k}$) and the resistance across the gap junctions ($R_g^{j,k}$):

$$R_i^{j,k} = R_c^{j,k} + R_g^{j,k}. \tag{S3}$$

Here, the gap junction resistance, $R_g^{j,k}$ is defined as

$$R_g^{j,k} = \frac{1}{G_g^{j,k}}, \tag{S4}$$

where $G_g^{j,k}$ (in mS) is the conductance through the gap junctions connecting cells j and k. Furthermore, the purely intracellular resistance, $R_c^{j,k}$, is

defined as $1/G_c^{j,k}$, where $G_c^{j,k}$ is given by (S1) for the purely intracellular space between the centers of cells j and k. In this case, the distance, l is given by $l_{j,k}$, the conductivity, σ, is given by the intracellular conductivity, σ_i, and the cross-sectional area, A, can be approximated by $\delta_i^{j,k} A_{j,k}$, where $\delta_i^{j,k}$ is the mean intracellular volume fraction of cell compartments j and k, and $A_{j,k}$ is, as above, the mean total cross-sectional area between the centers of compartments j and k, including both the intracellular and extracellular parts. Consequently, we get

$$R_c^{j,k} = \frac{1}{G_c^{j,k}} = \frac{l_{j,k}}{\delta_i^{j,k} A_{j,k} \sigma_i}. \tag{S5}$$

Inserting (S4) and (S5) into (S3), the conductance between cells j and k is defined as the reciprocal of $R_i^{j,k}$, i.e., by

$$G_i^{j,k} = \frac{1}{R_i^{j,k}} = \frac{1}{R_c^{j,k} + R_g^{j,k}} = \frac{1}{\frac{l_{j,k}}{\delta_i^{j,k} A_{j,k} \sigma_i} + \frac{1}{G_g^{j,k}}}. \tag{S6}$$

S2 Convergence of solutions

In order to investigate the numerical resolution required for the simulations, we perform simulations using a number of different resolutions and compare the conduction velocity (CV) and maximal upstroke velocity ($(\frac{dv}{dt})^{\max}$) for each resolution, h (Δt or Δx), to those computed for the finest considered resolution, $h^\star$. More specifically, we compute the error

$$\text{Error}(h) = \left(\frac{|\text{CV}_h - \text{CV}_{h^\star}|}{\text{CV}_{h^\star}} + \frac{|\left(\frac{dv}{dt}\right)_h^{\max} - \left(\frac{dv}{dt}\right)_{h^\star}^{\max}|}{\left(\frac{dv}{dt}\right)_{h^\star}^{\max}} \right) \cdot 100\%, \tag{S7}$$

where CV_h and $\left(\frac{dv}{dt}\right)_h^{\max}$ are the CV and maximal upstroke velocity, respectively, computed for the resolution h. The computation of the CV is as explained in the paper and the computation of the maximal upstroke velocity is as described below.

S2.1 Computation of the maximal upstroke velocity

In order to compute the maximal upstroke velocity, we record the membrane potential for each time step in the center cell for KNM and SKNM, and in a point in the center of the domain for BD and MD. From this recorded solution, the maximal upstroke velocity is computed as

$$\left(\frac{dv}{dt}\right)^{\max} = \max_n \left(\frac{v_{n+1} - v_{n-1}}{2\Delta t} \right), \tag{S8}$$

where v_n is the recorded membrane potential solution in time step n, and Δt is the size of the time step.

S2.2 Convergence of KNM and SKNM solutions for hiPSC-CMs

In Table S1, we investigate how the CV and maximal upstroke velocity computed using KNM and SKNM changes as the time step, Δt, used in the simulation is decreased. We report the CV and maximal upstroke velocity computed for a number of different values of Δt as well as the error (in percent) defined by (S7) with $h^\star = 0.001$ ms. We observe that a time step of $\Delta t = 0.02$ ms appears to be sufficient to achieve an error below 2%. In the KNM and SKNM simulations of hiPSC-CMs we therefore use a time step of $\Delta t = 0.02$ ms.

Δt (ms)	KNM CV (cm/s)	KNM $\left(\frac{dv}{dt}\right)^{\max}$ (V/s)	KNM Error	SKNM CV (cm/s)	SKNM $\left(\frac{dv}{dt}\right)^{\max}$ (V/s)	SKNM Error
1	3.33	23.44	36.2%	3.33	23.45	36.2%
0.2	3.57	20.73	15.4%	3.57	20.73	15.4%
0.1	3.60	19.22	6.5%	3.60	19.22	6.5%
0.02	3.73	18.81	1.0%	3.73	18.81	1.0%
0.01	3.75	18.77	0.7%	3.75	18.76	0.7%
0.002	3.76	18.78	0.2%	3.76	18.78	0.2%

Table S1: Conduction velocity (CV) and maximal upstroke velocity, $\left(\frac{dv}{dt}\right)^{\max}$, computed using different values of Δt in KNM and SKNM for a collection of hiPSC-CMs. The errors are computed by comparing the conduction velocity and maximal upstroke velocity to those computed using KNM or SKNM with $h^{\star} = 0.001$ ms (see (S7)).

S2.3 Convergence of BD and MD solutions for hiPSC-CMs

In Table S2 the CV and maximal upstroke velocity computed using BD and MD with a number of different spatial discretization parameters (Δx) are reported and compared to those computed for $\Delta x = 5$ μm. We observe that a spatial resolution of 10 μm is sufficient for the error to be less than 2%. Furthermore, in Table S3, we report similar results for a number of different time steps, Δt. Here, we observe that a time step of $\Delta t = 0.01$ ms is sufficient for the error to be below 2%. We therefore use a time step of $\Delta t = 0.01$ ms and spatial discretization of $\Delta x = 10$ μm for the BD and MD simulations.

Δx (μm)	BD CV (cm/s)	BD $\left(\frac{dv}{dt}\right)^{\max}$ (V/s)	BD Error	MD CV (cm/s)	MD $\left(\frac{dv}{dt}\right)^{\max}$ (V/s)	MD Error
200	4.25	22.95	37.0%	4.25	22.95	37.0%
100	3.95	21.25	19.9%	3.95	21.25	19.9%
50	3.88	18.85	4.8%	3.88	18.85	4.8%
20	3.82	18.65	2.2%	3.82	18.65	2.2%
10	3.78	18.45	0.02%	3.78	18.45	0.02%

Table S2: Conduction velocity (CV) and maximal upstroke velocity, $\left(\frac{dv}{dt}\right)^{\max}$, computed using different values of the spatial discretization parameter Δx in BD and MD for a collection of hiPSC-CMs. The errors are computed by comparing the conduction velocity and maximal upstroke velocity to those computed using BD or MD with $h^{\star} = 5$ μm (see (S7)). All the simulations in this table are performed using a time step of $\Delta t = 0.001$ ms.

Δt (ms)	BD CV (cm/s)	BD $\left(\frac{dv}{dt}\right)^{\max}$ (V/s)	BD Error	MD CV (cm/s)	MD $\left(\frac{dv}{dt}\right)^{\max}$ (V/s)	MD Error
1	3.33	14.12	35.2%	3.33	14.12	35.2%
0.2	3.57	16.96	13.5%	3.57	16.96	13.5%
0.1	3.60	17.17	11.5%	3.60	17.16	11.5%
0.02	3.74	18.12	2.8%	3.74	18.11	2.8%
0.01	3.76	18.26	1.5%	3.76	18.26	1.5%
0.002	3.77	18.43	0.2%	3.77	18.43	0.2%

Table S3: Conduction velocity (CV) and maximal upstroke velocity, $\left(\frac{dv}{dt}\right)^{\max}$, computed using different values of Δt in BD and MD for a collection of hiPSC-CMs. The errors are computed by comparing the conduction velocity and maximal upstroke velocity to those computed using BD or MD with $h^{\star} = 0.001$ ms (see (S7)). All the simulations in this table are performed using a spatial discretization of $\Delta x = 5$ μm.

S2.4 Convergence of KNM and SKNM for pancreatic β cells

In Table S4, we report the CV, maximal upstroke velocity and the error of SKNM and KNM simulations for pancreatic β cells for different values of Δt. Again, we observe that a time step of $\Delta t = 0.02$ ms seems to be sufficient to ensure an error below 2%. We therefore use $\Delta t = 0.02$ ms for the KNM and SKNM β cell simulations in this study.

Δt (ms)	KNM CV (cm/s)	KNM $\left(\frac{dv}{dt}\right)^{\max}$ (V/s)	KNM Error	SKNM CV (cm/s)	SKNM $\left(\frac{dv}{dt}\right)^{\max}$ (V/s)	SKNM Error
2	0.0239	0.385	15.1%	0.0239	0.385	15.1%
1	0.0242	0.366	8.0%	0.0242	0.366	8.0%
0.2	0.0245	0.350	3.7%	0.0245	0.350	3.7%
0.1	0.0245	0.348	3.2%	0.0245	0.348	3.2%
0.02	0.0243	0.340	0.1%	0.0243	0.340	0.1%
0.01	0.0243	0.340	0.0%	0.0243	0.340	0.0%

Table S4: Conduction velocity (CV) and maximal upstroke velocity, $\left(\frac{dv}{dt}\right)^{\max}$, computed using different values of Δt in KNM and SKNM for a collection of β cells. The errors are computed by comparing the conduction velocity and maximal upstroke velocity to those computed using KNM or SKNM with $h^{\star} = 0.005$ ms (see (S7)).

S3 SKNM based on negligible extracellular potential, SKNM($u_e = 0$)

In the derivation of KNM (see [2]), Kirchhoff's current law is first applied for each cell k in the tissue. This results in the equation

$$C_m \frac{dv^k}{dt} = \frac{1}{A_m^k} \sum_{j \in N_k} I_i^{j,k} - I_{\text{ion}}^k(v^k, s^k), \tag{S9}$$

where

$$I_i^{j,k} = G_i^{j,k}(u_i^j - u_i^k), \tag{S10}$$

and

$$v^k = u_i^k - u_e^k. \tag{S11}$$

To derive KNM, we additionally need to apply Kirchhoff's current law for the extracellular compartment associated with each cell. However, in some cases it could be appropriate to instead assume that the extracellular potential, u_e^k, surrounding the cells is negligible (close to 0). In that case, (S11) gives $v^k = u_i^k$, which can be inserted into (S10) and (S9) to yield

$$C_m \frac{dv^k}{dt} = \frac{1}{A_m^k} \sum_{j \in N_k} G_i^{j,k}(v^j - v^k) - I_{\text{ion}}^k(v^k, s^k), \tag{S12}$$

$$\frac{ds^k}{dt} = F_k(s^k, v^k), \tag{S13}$$

where we have also included the equation (S13) for the additional state variables of the membrane model. We observe that the model (S12)–(S13) is the same as SKNM, except that the factor $\frac{\lambda}{1+\lambda}$ is excluded from (S12) (i.e., the factor is set to 1). Note that we would also arrive at the same model if we assumed that $G_e^{j,k} \gg G_i^{j,k}$. In that case, λ would be very large and the factor $\frac{\lambda}{1+\lambda}$ in SKNM would be very close to 1. We here refer to the alternative model (S12)–(S13) resembling SKNM as SKNM($u_e = 0$). In the next subsections, we will investigate how well this further simplification of KNM approximates KNM in the two applications investigated in the main manuscript.

S3.1 SKNM($u_e = 0$) for a collection of β-cells

In Figure S1, we have computed the conduction velocity for several choices of the gap junction variation, γ, and the extracellular volume fraction for both KNM, SKNM and SKNM($u_e = 0$) in the case of a collection of β cells. We observe that KNM, SKNM and SKNM($u_e = 0$) all provide identical solutions in terms of conduction velocity for these examples.

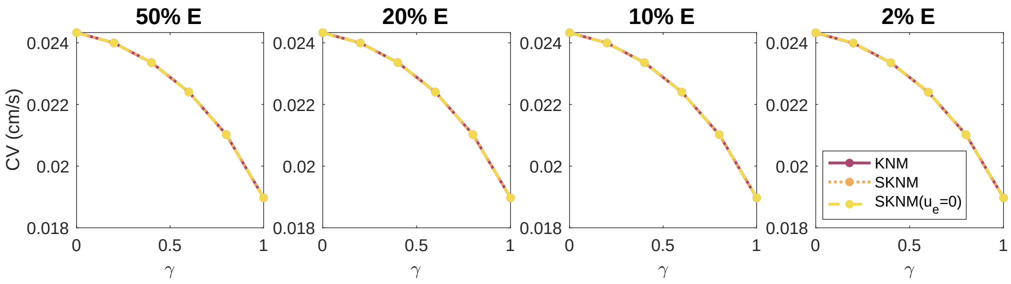

Figure S1: Comparison of the conduction velocity (CV) computed using KNM, SKNM and SKNM assuming a negligible extracellular potential, SKNM($u_e = 0$), for a collection of pancreatic β cells with an increasing gap junction conductance variation, γ. We consider the cases of 50%, 20%, 10%, and 2% extracellular space as indicated in the panel titles.

S3.2 SKNM($u_e = 0$) for a collection of hiPSC-CMs

In Figure S1, we observed that both SKNM and the version of SKNM where the extracellular potential is assumed to be negligible, i.e., SKNM($u_e = 0$), provided very good approximations of KNM for a collection of pancreatic β cells. In Figure S2, however, we observe that the assumption of negligible extracellular potential does not provide an equally good approximation for a collection of hiPSC-CMs. For a large extracellular volume percentage (50%), the conduction velocities computed for SKNM($u_e = 0$) seem quite close to those computed for KNM and SKNM, but as the extracellular volume percentage is decreased, SKNM($u_e = 0$) gradually approximate KNM less accurately.

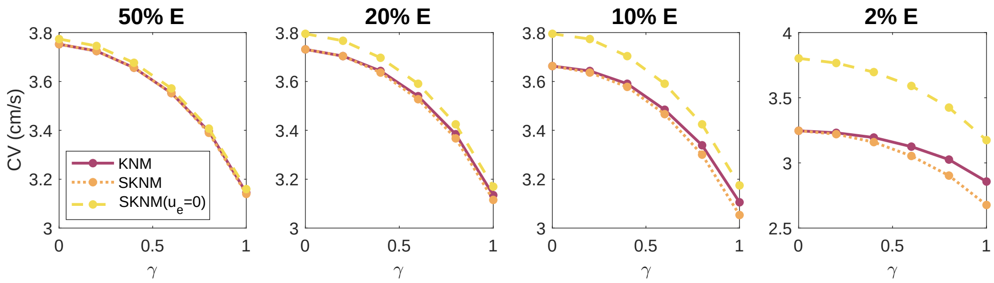

Figure S2: Comparison of the conduction velocity (CV) computed using KNM, SKNM, and SKNM with the assumption of a negligible extracellular potential, SKNM($u_e = 0$), for a collection of hiPSC-CMs with an increasing gap junction conductance variation, γ. We consider the cases of 50%, 20%, 10%, and 2% extracellular space as indicated in the panel titles.

S4 A special case where the discretized BD and MD are equal to KNM and SKNM

In this section, we will show that in the special case where a collection of cells is organized in a structured grid and BD and MD are spatially discretized using a finite difference approximation with resolution equal to the cell size, then the spatially discretized BD and MD equal the KNM and SKNM, respectively. Furthermore, if the SKNM assumption holds as well, then the algebraic equations defining all four models coincide.

S4.1 Modeling setup

For simplicity, we consider the 2D versions of the BD and MD models, corresponding to a 2D collection of cells in KNM and SKNM. We assume that each cell has the maximal length of l_x in the x-direction, l_y in the y-direction and l_z in the z-direction, and that the cells are organized in a structured grid like, e.g., illustrated in Figures 1–2 and Figures 10–11 in the paper. Note that we do not assume that a cell makes up the entire volume $l_x l_y l_z$. This volume is also assumed to contain some extracellular space. Rather, l_x, l_y and l_z are the maximal cell lengths in each spatial direction, corresponding to the distance between the points where the cell connections in each direction are located.

S4.2 Finite difference approximation of BD equal to KNM

Let us first recall the definition of the bidomain model:

$$C_m \frac{\partial v}{\partial t} = \chi^{-1} \left(\nabla \cdot (M_i \nabla v) + \nabla \cdot (M_i \nabla u_e) \right) - I_{\text{ion}}(s, v), \tag{S14}$$

$$0 = \nabla \cdot (M_i \nabla v) + \nabla \cdot ((M_i + M_e) \nabla u_e), \tag{S15}$$

$$\frac{ds}{dt} = F(s, v), \tag{S16}$$

with

$$M_i = \begin{pmatrix} M_i^x & 0 \\ 0 & M_i^y \end{pmatrix}, \qquad M_e = \begin{pmatrix} M_e^x & 0 \\ 0 & M_e^y \end{pmatrix}, \tag{S17}$$

and

$$M_i^x = \frac{\delta_i \sigma_i}{1 + \delta_i \sigma_i \frac{l_y l_z}{l_x G_g}}, \qquad M_i^y = \frac{\delta_i \sigma_i}{1 + \delta_i \sigma_i \frac{l_x l_z}{l_y G_g}}, \tag{S18}$$

$$M_e^x = M_e^y = \delta_e \sigma_e. \tag{S19}$$

See the paper for definitions and units for each of the model parameters and variables.

We wish to spatially discretize this model with a resolution equal to the cell size. In other words, we use the spatial discretization parameters $\Delta x = l_x$ and $\Delta y = l_y$ in the x- and y-directions, respectively. In addition, we let the grid points correspond to the center of the cells. We let p denote the cell number in the x-direction, which equals the grid point number in the x-direction and q denote the cell (and grid point) number in the y-direction. A finite difference discretization of (S14)–(S16) for points not on the boundary then reads

$$\begin{aligned}
C_m \frac{dv^{p,q}}{dt} = \chi^{-1} \Big(& \tfrac{M_i^{x,p+1/2,q}(v^{p+1,q}-v^{p,q}) - M_i^{x,p-1/2,q}(v^{p,q}-v^{p-1,q})}{l_x^2} \\
&+ \tfrac{M_i^{y,p,q+1/2}(v^{p,q+1}-v^{p,q}) - M_i^{y,p,q-1/2}(v^{p,q}-v^{p,q-1})}{l_y^2} \\
&+ \tfrac{M_e^{x,p+1/2,q}(u_e^{p+1,q}-u_e^{p,q}) - M_e^{x,p-1/2,q}(u_e^{p,q}-u_e^{p-1,q})}{l_x^2} \\
&+ \tfrac{M_e^{y,p,q+1/2}(u_e^{p,q+1}-u_e^{p,q}) - M_e^{y,p,q-1/2}(u_e^{p,q}-u_e^{p,q-1})}{l_y^2} \Big) - I_{\text{ion}}(v^{q,p}, s^{q,p})
\end{aligned} \tag{S20}$$

$$\begin{aligned}
0 = & \tfrac{M_i^{x,p+1/2,q}(v^{p+1,q}-v^{p,q}) - M_i^{x,p-1/2,q}(v^{p,q}-v^{p-1,q})}{l_x^2} \\
&+ \tfrac{M_i^{y,p,q+1/2}(v^{p,q+1}-v^{p,q}) - M_i^{y,p,q-1/2}(v^{p,q}-v^{p,q-1})}{l_y^2} \\
&+ \tfrac{(M_i^{x,p+1/2,q}+M_e^{x,p+1/2,q})(u_e^{p+1,q}-u_e^{p,q}) - (M_i^{x,p-1/2,q}+M_e^{x,p-1/2,q})(u_e^{p,q}-u_e^{p-1,q})}{l_x^2} \\
&+ \tfrac{(M_i^{y,p,q+1/2}+M_e^{y,p,q+1/2})(u_e^{p,q+1}-u_e^{p,q}) - (M_i^{y,p,q-1/2}+M_e^{y,p,q-1/2})(u_e^{p,q}-u_e^{p,q-1})}{l_y^2}
\end{aligned} \tag{S21}$$

$$\frac{ds^{p,q}}{dt} = F(s^{p,q}, v^{p,q}), \tag{S22}$$

where $v^{p,q}$ and $u_e^{p,q}$ are the finite difference approximations of v and u_e, respectively, in the grid point corresponding to the center of cell (p,q). Moreover, as an example, $M_i^{x,p+1/2,q}$ is the value of M_i^x between the centers of cell (p,q) and cell $(p+1,q)$.

S4.2.1 Rewriting the discretization in terms of general neighbors

In order to compare this scheme to KNM, we now let the cell (p,q) be denoted as cell number k. Furthermore, if we, for instance, let cell $(p+1,q)$ be denoted

by j, then according to (S18), $M_i^{x,p+1/2,q}$ is given by

$$M_i^{x,p+1/2,q} = \frac{\delta_i^{j,k}\sigma_i}{1 + \delta_i^{j,k}\sigma_i \frac{A_{j,k}}{l_{j,k}G_g^{j,k}}}. \tag{S23}$$

Following similar arguments for each instance of M_i and M_e appearing in (S20)–(S21) and defining

$$M_i^{j,k} = \frac{\delta_i^{j,k}\sigma_i}{1 + \delta_i^{j,k}\sigma_i \frac{A_{j,k}}{l_{j,k}G_g^{j,k}}} \tag{S24}$$

$$M_e^{j,k} = \delta_e^{j,k}\sigma_e, \tag{S25}$$

the discrete system (S20)–(S22) can be written as

$$C_m \frac{dv^k}{dt} = \chi^{-1} \sum_{j\in N_k} \left\{ \frac{M_i^{j,k}}{l_{j,k}^2}(v^j - v^k) + \frac{M_e^{j,k}}{l_{j,k}^2}(u_e^j - u_e^k) \right\} - I_{\text{ion}}(v^k, s^k), \tag{S26}$$

$$0 = \sum_{j\in N_k} \frac{M_i^{j,k}}{l_{j,k}^2}(v^j - v^k) + \sum_{j\in N_k} \frac{M_i^{j,k} + M_e^{j,k}}{l_{j,k}^2}(u_e^j - u_e^k), \tag{S27}$$

$$\frac{ds^k}{dt} = F(s^k, v^k), \tag{S28}$$

where N_k is the collection of all the neighbors of cell k. For the notation (p, q) for cell k, the neighbors are cells $(p + 1, q)$, $(p - 1, q)$, $(p, q + 1)$ and $(p, q - 1)$.

We assume that the geometry of all cells is the same. Therefore, the membrane surface to volume ratio is given by

$$\chi = \frac{A_m}{l_x l_y l_z}, \tag{S29}$$

where A_m is the membrane area of a cell and $l_x l_y l_z$ is the total volume (both intracellular and extracellular) of each compartment. We also note that for any cell neighbours, j and k, the volume $l_x l_y l_z = A_{j,k} l_{j,k}$, where $A_{j,k}$ is the total cross-sectional area between the centers of cell j and k including both the intracellular and extracellular spaces, and $l_{j,k}$ is the distance between the cell centers. Inserting this in (S26), we get

$$C_m \frac{dv^k}{dt} = \frac{1}{A_m} \sum_{j\in N_k} \left\{ \frac{A_{j,k}M_i^{j,k}}{l_{j,k}}(v^j - v^k) + \frac{A_{j,k}M_e^{j,k}}{l_{j,k}}(u_e^j - u_e^k) \right\} - I_{\text{ion}}(v^k, s^k).$$

Using (S24) and (S25), we observe that

$$\frac{A_{j,k}M_i^{j,k}}{l_{j,k}} = \frac{A_{j,k}}{l_{j,k}} \frac{\delta_i^{j,k}\sigma_i}{1+\delta_i^{j,k}\sigma_i \frac{A_{j,k}}{l_{j,k}G_g^{j,k}}} = \frac{A_{j,k}\delta_i^{j,k}\sigma_i}{l_{j,k}+\delta_i^{j,k}\sigma_i \frac{A_{j,k}}{G_g^{j,k}}}$$

$$= \frac{1}{\frac{l_{j,k}}{\delta_i^{j,k}A_{j,k}\sigma_i} + \frac{1}{G_g^{j,k}}} = G_i^{j,k}, \tag{S30}$$

$$\frac{A_{j,k}M_e^{j,k}}{l_{j,k}} = \frac{A_{j,k}}{l_{j,k}}\delta_e^{j,k}\sigma_e = G_e^{j,k}, \tag{S31}$$

which equals the KNM parameters $G_e^{j,k}$ and $G_i^{j,k}$ defined in (S2) and (S6).

Now, multiplying both sides of (S27) by $l_x l_y l_z$ and inserting (S30) and (S31) into (S26)–(S28), we get the following discrete version of BD,

$$C_m\frac{dv^k}{dt} = \frac{1}{A_m}\sum_{j\in N_k}\left\{G_i^{j,k}(v^j - v^k) + G_e^{j,k}(u_e^j - u_e^k)\right\} - I_{\text{ion}}(v^k, s^k), \tag{S32}$$

$$0 = \sum_{j\in N_k} G_i^{j,k}(v^j - v^k) + \sum_{j\in N_k}(G_i^{j,k} + G_e^{j,k})(u_e^j - u_e^k), \tag{S33}$$

$$\frac{ds^k}{dt} = F(s^k, v^k), \tag{S34}$$

which is exactly the same as KNM with a constant value of A_m (see the paper).

S4.2.2 Boundary conditions

So far, we have only considered the discretized BD in the grid points that are not located on the boundary of the domain. However, we may also discretize the boundary conditions of BD such that the resulting system of equations equals KNM also at the boundary. We consider homogenous Neumann boundary conditions for both v and u_e on the entire boundary except for a homogeneous Dirichlet boundary condition for u_e in a corner.

To make sure that the discrete version of BD equals KNM, the boundary conditions for u_e should be discretized in the same manner as what we use for the boundary conditions for u_e in KNM, for example a first order finite difference approximation like we have done in the computations in the paper. We should also use a first order difference for the boundary conditions for v. For example, for the grid point in the upper right corner, the homogenoeus Neumann boundary conditions state that the normal derivative of v should be zero in the positive x- and y-directions. This can be approximated by the

first order finite differences

$$\frac{v^{p+1,q} - v^{p,q}}{l_x} = 0 \tag{S35}$$

$$\frac{v^{p,q+1} - v^{p,q+1}}{l_x} = 0, \tag{S36}$$

where $v^{p+1,q}$ and $v^{p,q+1}$ are imagined grid points located outside of the actual cell collection. Inserting this in (S20)–(S22) amounts to some of the terms on the right-hand side of (S20)–(S22) being set to zero. More specifically, the terms corresponding to currents to the upper and right neighbours are set to zero. But since there are no upper and right neighbours for the upper right corner cell, the currents between the cell and all the *actual* neighbours are still included in (S20)–(S22). Consequently, the reorganized system still reads (S32)–(S34), which is equal to KNM. This similarly holds for the remaining boundary points as well.

S4.2.3 Finite difference approximation of MD equal to SKNM

Following the same approach as above it can also be shown that a similarly discretized version of MD equals SKNM.

S4.3 Equal algebraic equations for KNM, SKNM, BD and MD

When the SKNM assumption,

$$G_e^{j,k} = \lambda G_i^{j,k}, \tag{S37}$$

holds, we know from the paper that SKNM and KNM are equal. Since the special discrete version of BD considered above is equal to KNM, it must therefore also be equal to SKNM in this case. Similarly, since a similar discrete version of MD is equal to SKNM, this special discrete version of MD must also be equal to KNM and the discrete BD. Consequently, in this special case, the algebraic equations would be equal for all four of the considered models.

References

[1] Robert Plonsey and Roger C Barr. *Bioelectricity, A Quantitative Approach*. Springer, 2007.

[2] Karoline H. Jæger and Aslak Tveito. Efficient, cell-based simulations of cardiac electrophysiology; the Kirchhoff Network Model (KNM). *npj Systems Biology and Applications*, 9:25, 2023.
